# Lessons from RatA: Why the Basics in Molecular Biology Are Still Crucial!

**DOI:** 10.3390/ijms26073100

**Published:** 2025-03-27

**Authors:** Michel Fasnacht, Denise Schratt, Isabella Moll

**Affiliations:** 1 Max Perutz Labs, Vienna Biocenter Campus (VBC), Dr.-Bohr-Gasse 9/Vienna Biocenter 5, 1030 Vienna, Austria; 2 University of Vienna, Max Perutz Labs, Department of Microbiology, Immunobiology and Genetics, Dr.-Bohr-Gasse 9/Vienna Biocenter 5, 1030 Vienna, Austria

**Keywords:** RatA, PasT, *Escherichia coli*, genome annotation, TA systems

## Abstract

Since the first bacterial genomes were sequenced and annotated over 25 years ago, sequencing technologies have rapidly advanced in both speed and cost efficiency. To date, over two million annotated bacterial genomes have been deposited in the National Center for Biotechnology Information (NCBI) database. Yet, there are many genes with unknown functions and, furthermore, conflicting results have been published for many investigated genes. One example is the *ratA* (or *pasT*) gene from *Escherichia coli* (*E. coli*) K-12 strains. Initially identified as a ribosome-targeting toxin, later studies described RatA as the bacterial homolog of the mitochondrial Coq10 protein and, therefore, beneficial for *E. coli* cells during aerobic growth. This study shows that these conflicting results originated from a mis-annotation of the start codon in the genomic sequence. Overexpression of the *ratA* gene as currently annotated leads to the synthesis of two RatA protein variants, a toxic and a non-toxic one. This study further identifies the endogenous *ratA* promoter and shows that only the shorter, non-toxic variant of RatA is synthesized during different growth phases specifically under aerobic conditions. Our findings thereby not only solidify the role of RatA in *E. coli*, but also demonstrate the importance of first validating the basics of molecular biology when investigating a previously poorly described gene, even in times of advanced high-throughput techniques.

## 1. Introduction

The Gram-negative bacterium *E. coli* is an intensively well-studied, facultative, anaerobic, natural colonizer of the human gut with many clinically relevant strains [1]. Among the different *E. coli* strains, the K-12 strains remain among the preferred model organisms for researchers. Accordingly, the sequencing and annotation of the complete *E. coli* K-12 genome in 1997 represented a pivotal breakthrough [2]. Since then, over two million annotated bacterial genomes have been deposited in the NCBI database [3]. In addition, bioinformatic databases have emerged to curate this enormous amount of data. For instance, EcoCyc aims to describe the complete molecular catalog of an *E. coli* K-12 cell and summarizes the available research on individual genes and their respective gene products [4]. Despite these ongoing efforts, many genes with unknown functions remain elusive. In other cases, conflicting results have been published on the physiological roles of specific genes and their respective gene products, including the ribosome association toxin A (RatA).

*ratA* (formerly *yfjG*) is encoded together with *ratB* (formerly *yfjF*) on the *E. coli* K-12 chromosome in the bi-cistronic operon *ratAB* (Figure S1A). RatA was first identified as a potential toxin in a broader screen of putative toxin–antitoxin (TA) pairs [5] (for a review of TA systems, see [6]). In a follow-up study, Zhang and Inouye further characterized RatA in the *E. coli* K-12 strain BW25113 as a ribosome-targeting toxin that binds to the 50S subunit and thereby inhibits ribosomal subunits association [7].

A similar toxic effect was described in a later study on *pasT* [8]. PasT is the uropathogenic *E. coli* (UPEC) homolog of RatA, with the *pasT* gene differing from *ratA* in 21 silent and 2 non-silent mutations (resulting in the amino acid changes S90N and D111E, Figure S1B). As Zhang and Inouye did before, the authors of this study described a toxic effect upon the overexpression of *pasT*/*ratA* that was dependent on the presence of the annotated N-terminus. Furthermore, they showed that the subsequent overexpression of *pasI*, the *ratB* homolog, removed the toxic effect of *pasT* overexpression and allowed growth to resume normally, thus indicating that *pasTI*/*ratAB* are true antagonistic proteins [8]. However, *ratB* deletion strains are viable [9], arguing against *pasTI*/*ratAB* being a classical TA system. In addition, the presence of PasT was non-toxic for the UPEC strain CFT073, as evidenced by the observation that a pronounced small-colony phenotype upon the deletion of the *pasTI* operon was rescued by low-level ectopic *pasT* expression [8].

The same small-colony phenotype in a *pasTI*-depleted CFT073 strain was further confirmed in a more recent study by Fino et al. [10]. However, using spot assays, the authors did not identify the same toxic effect upon the overproduction of PasT/RatA, as previously observed [7,8], which they attributed to lower expression levels. Instead, they identified PasT as likely to be the bacterial equivalent of its mitochondrial homolog Coq10, a lipid chaperone for ubiquinone in the electron transport chain [10]. Consistently, a loss of PasT leads to a slightly reduced membrane potential and the previously described small-colony phenotype was only observed under aerobic conditions [10].

In summary, two opposing roles have been described for RatA/PasT in *E. coli*. Under aerobic growth conditions, a low-level expression of *ratA* or *pasT* has been indicated to support the electron transport chain and increase the infectivity of UPEC cells. On the other hand, the overexpression of *ratA* or *pasT* has been shown to have a toxic effect on cell growth and translation. In this study, we show that upon the overexpression of the *ratA* gene as currently annotated in the K-12 reference genome, two variants of RatA are produced, originating from differential translation initiation at distinct start codons. We further demonstrate that only the full-length RatA is toxic. Using an endogenous promoter system under aerobic conditions, we validate that under different growth conditions, from the early exponential into the late stationary phase, only the shorter, non-toxic variant of RatA is produced. Further analysis of the corresponding promoter region reveals that the open reading frame (ORF) of *ratA* is currently mis-annotated in the reference genome sequence, a problem that might be more widespread in genes with unknown functions and not restricted to *ratA*. Therefore, this study highlights the importance of first validating the basics of molecular biology when investigating a previously poorly described gene, even in times of advanced high-throughput techniques.

## 2. Results

### 2.1. Overexpression of ratA Leads to the Production of Two RatA Variants

**Figure 1:**
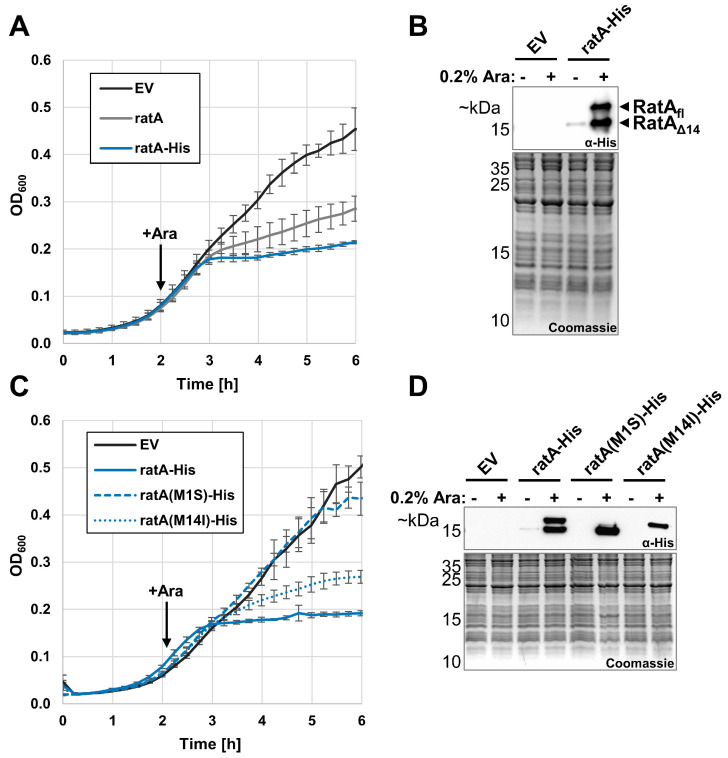
Overexpression of *ratA* leads to the generation of both a toxic and a non-toxic variant of RatA. (**A**) *E. coli* strain BW25113 was transformed with plasmid pBAD–ratA (ratA, grey solid line) or pBAD–ratA–His (ratA–His, blue solid line) carrying the wild-type *ratA* gene or a 3′-terminal His-tagged *ratA* gene under arabinose inducible promoter control. Growth in LB medium at 37 °C was monitored in a 96-well plate before and after the addition of arabinose to induce the overexpression of both genes. As a negative control, cells carrying an empty vector (EV, solid black line) were treated equally. The average of 3 biological replicates is shown with the standard deviation indicated by grey bars. (**B**) Representative western blot analysis of samples harvested from BW25113 cells carrying the empty vector or pBAD–ratA–His plasmid before or 1 h after the addition of arabinose. The employed primary antibody is indicated in the immunoblot box (top) and a Coomassie gel was used as loading control (bottom). (**C**,**D**) As in (**A**,**B**), but with the addition of cells carrying the plasmids pBAD–ratA(M1S)–His (dashed blue line) or pBAD–ratA(M14I)–His (dotted blue line) containing mutations of the methionine codons at position 1 or position 14 of the annotated ORF of *ratA*, respectively.

As our lab has previously been interested in ribosome-targeting toxins [11,12], we initially set out to confirm the results from Zhang and Inouye [7]. Therefore, employing the *E. coli* K-12 strain BW25113, we first overexpressed the wild-type *ratA* gene in the early exponential phase. Consistent with previous results [7], a post-induction growth arrest was observed after an initial delay (Figure 1A, grey line). Furthermore, reduced cell divisions upon overexpression were still observed when a 3′-terminal His-tag sequence was cloned in-frame to the *ratA* gene (Figure 1A, blue line). Surprisingly, a western blot analysis performed to confirm the synthesis of the RatA–His fusion protein revealed two signals after the addition of the inducer (Figure 1B). To the best of our understanding, Zhang and Inouye previously only detected a single protein in their western blot analyses by employing an N-terminal His-tag [7], whereas Norton and Mulvey, employing a C-terminal FLAG-tag on the RatA-homolog PasT, likely detected a similar double band [8]. Therefore, we concluded that the two observed proteins differ in their N-termini.

### 2.2. Full-Length ratA Is Responsible for the Observed Toxic Effect

Closer analysis of the *ratA* gene reveals the presence of three in-frame methionine (Met) codons close to the start of the annotated ORF (Figure S1). Previously, Norton and Mulvey established a domain-dependency of the toxic effect of PasT using a series of *pasT* gene truncations [8]. Only full-length PasT originating from the first annotated start codon inhibited growth, whereas the increased synthesis of a truncated PasT variant lacking the first 13 amino acids had no toxic effect on the cells. However, a leaky expression of the shortened gene was sufficient to complement the small-colony phenotype observed in the *pasTI* knockout strain [8]. Taking these observations together, we hypothesized that the two signals in Figure 1B originate from differential initiations at the Met1 and Met14 codons, respectively. To test this hypothesis, we overexpressed a *ratA(M14I)*–*His* mutant lacking the potential start codon inside of the annotated ORF. Consistent with our hypothesis, a toxic effect, as evidenced by the arrested growth of the cells, was still observed, but only the larger protein variant was detected by immunoblotting (Figure 1C,D). In contrast, when overexpressing a *ratA(M1S)*–*His* mutant lacking the first annotated start codon, no toxic effect was observed and only the shorter protein was detected by western blot analysis (Figure 1C,D). To further solidify these findings, we repeated the overexpression of the wild-type *ratA* gene with a 3′-terminal Strep-tag sequence added in-frame, purified both synthesized protein variants by their Strep-tag, and analyzed their intact mass by LC-MS (Figure S2). Consistent with our findings above, the mass spectrometry analysis indicated with a mass error of −0.5 Da that the faster-migrating protein corresponded to a RatA variant consisting of the amino acids 15–166 (Met14 excised, Strep-tag still present, no post-translational modification) and with a mass error of −0.4 Da that the slower-migrating protein corresponded to the full-length RatA entailing amino acids 1–166 (Met1 not excised, Strep-tag still present, no post-translational modification). Therefore, we termed the two variants RatA_Δ14_ and RatA_fl_, respectively.

### 2.3. RatA_fl_ Does Not Exclusively Target the 50S Subunit

**Figure 2:**
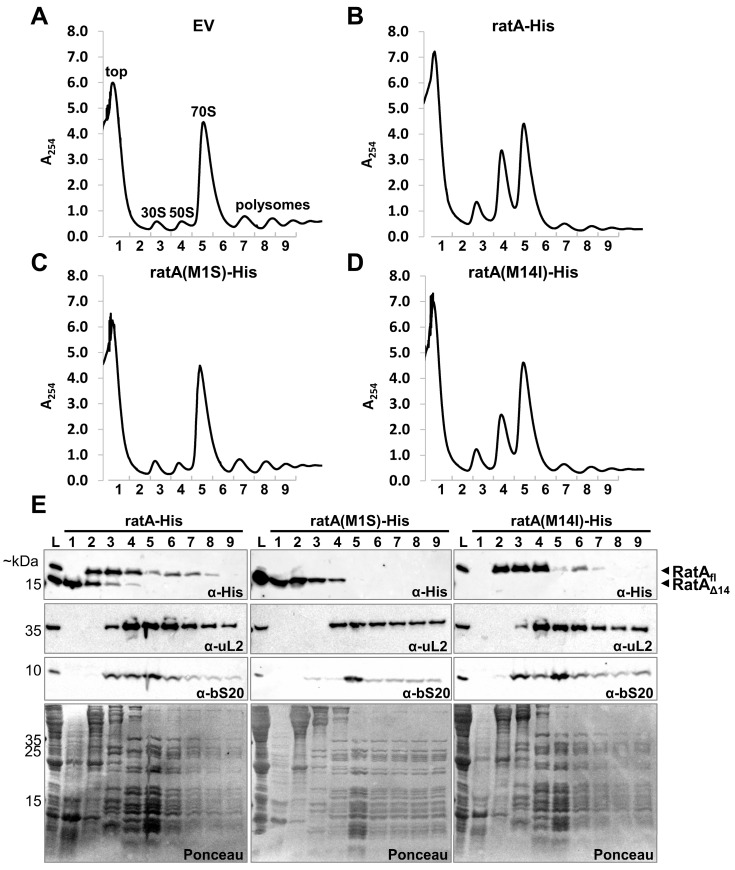
Toxic RatA_fl_ does not exclusively target the 50S subunit. Cell extract was prepared and fractionated on 10–40% sucrose gradients from BW25113 cells carrying the empty vector (EV, (**A**)) or plasmids pBAD–ratA–His (**B**), pBAD–ratA(M1S)–His (**C**), or pBAD–ratA(M14I)–His (**D**) 1.5 h after the addition of arabinose to induce the overexpression of the respective genes. The elution of different ribosomal particles was monitored (A_254_) and indicated fractions on the x-axis were collected for western blot analysis in (**E**) together with a total lysate sample (L) as input control. Localization of RatA_fl_ and RatA_Δ14_ was monitored (top). To control for the proper separation of the ribosomal subunits, localization of both a large (uL2) and small subunit (bS20) ribosomal protein was monitored. As an additional loading control, a Ponceau stain of the respective membranes was performed (bottom).

As a toxic effect on growth was only observed when RatA_fl_ was synthesized, we speculated that only the RatA_fl_ variant targets the 50S subunit, as previously described [7]. To confirm this hypothesis, we repeated the overexpression of the wild-type *ratA*–*His* gene together with the *ratA(M1S)–His* and *ratA(M14I)–His* mutants and fractionated their corresponding cell lysates on sucrose gradients. Compared to the empty vector control in Figure 2A, the overexpression of the wild-type gene in Figure 2B resulted in a similar phenotype as previously published [7]. As expected, the same reduction in polysomes and increase in free subunits were observed when the *ratA(M14I)*–*His* mutant was overexpressed (Figure 2D). Conversely, no such phenotype was observed upon the overexpression of the non-toxic *ratA(M1S)*–*His* mutant (Figure 2C). Finally, to investigate the binding of the two protein variants to the ribosome, the presence of either RatA_fl_ or RatA_Δ14_ in the collected sucrose fractions was examined by western blot analysis. As shown in Figure 2E, the two RatA variants did not sediment equally. While RatA_Δ14_ was mostly detected in the top fraction where free proteins are found, RatA_fl_ was completely absent in this fraction. Instead, RatA_fl_ was present in the fractions before and during the elution of the 30S and 50S subunits, and to a minor extent in the 70S and polysomes fractions. No clear association with the 50S subunit was observed as previously described [7], raising the question of whether RatA_fl_ truly is a ribosome-targeting toxin or whether the observed increased abundance of free ribosomal subunits is instead an indirect effect of *ratA* overexpression.

### 2.4. Only ratA_Δ14_ Is Detected in Unstressed Cells

**Figure 3:**
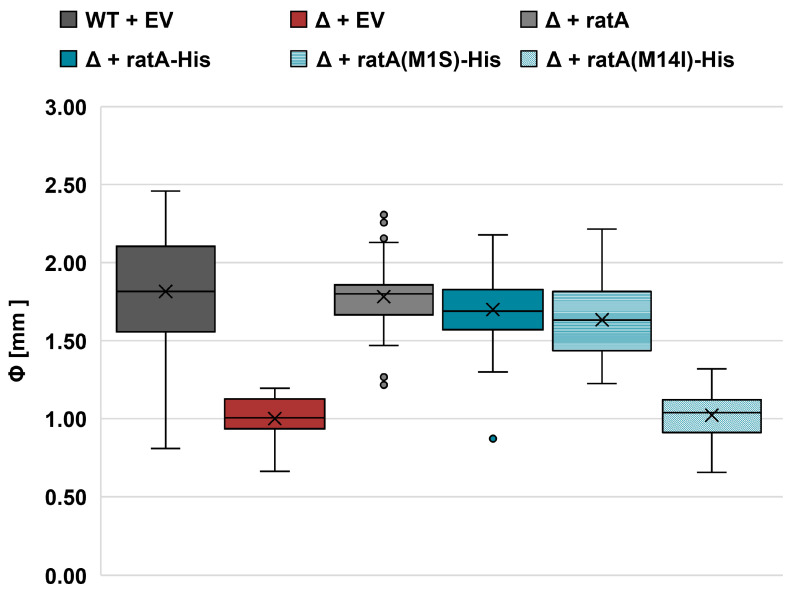
The internal methionine codon Met14 is required to complement the small-colony phenotype upon genomic deletion of *ratA*. Box and whisker plot of colony sizes after 20 h of growth on LB plates at 37 °C (n = 50). Black horizontal lines represent the exclusive median, whereas mean values are marked by an x. The wild-type BW25113 (WT) or corresponding *ratA* deletion strain (Δ) was transformed with either an empty vector control (EV) or the low-copy plasmid pZS*2-ratA carrying the wild-type *ratA* gene under endogenous promoter control (ratA). The same plasmid was further manipulated to introduce a 3′-terminal His-tag sequence (ratA–His) and to mutate the first annotated start codon of the ORF (M1S) or the internal methionine codon at position 14 (M14I).

To gain further insight into the physiological role of RatA_fl_ and/or RatA_Δ14_, the *ratA* gene was cloned into a low-copy plasmid system under presumed endogenous promoter control, as described in the Materials and Methods section. To avoid interference from the chromosomally encoded copy, a *ratA* deletion strain was employed. Consistent with previously published results [8,10], a small-colony phenotype was observed upon the deletion of the *ratA* gene (Figure 3, solid red box). As expected, complementation with the pZS*2-ratA plasmid completely restored the wild-type colony size (Figure 3, solid grey box). Furthermore, to facilitate the downstream detection of RatA, we inserted a His-tag sequence at the 3′-end of the *ratA* gene on the plasmid, which did not interfere with the complementation of the small-colony phenotype (Figure 3, solid blue boxes).

**Figure 4:**
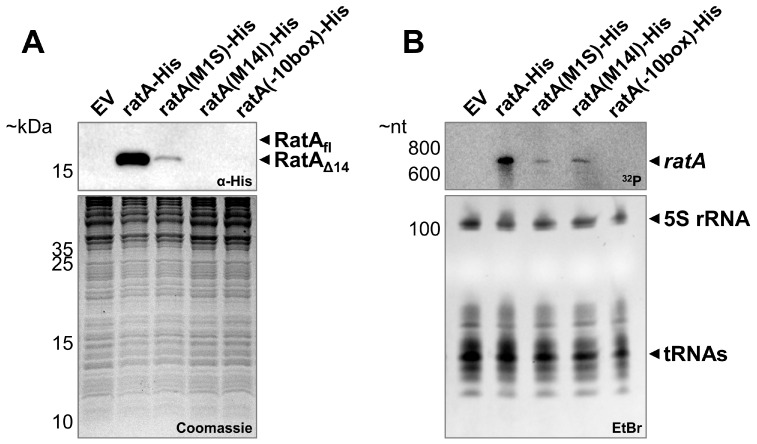
Only RatA_Δ14_, no RatA_fl_, is synthesized in exponentially growing cells. Samples were simultaneously harvested from exponentially growing cells incubated in LB medium at 37 °C for both western blot (**A**) and northern blot analysis (**B**). The BW25113(Δ*ratA*) deletion strain transformed with either an empty vector control (EV) or the low-copy plasmid pZS*2–ratA–His carrying the wild-type *ratA* gene with a 3′-terminal His-tag sequence under endogenous promoter control (ratA–His) was employed. The pZS*2–ratA–His plasmid was further manipulated to mutate the first annotated start codon of the ORF (M1S), the internal methionine codon at position 14 (M14I), or the −10 box of the predicted σ^70^ promoter (−10box). For the western blot analysis, the employed primary antibody is indicated in the immunoblot box (top) and a Coomassie gel was used as loading control (bottom). For the northern blot analysis of total RNA samples, a ^32^P-labelled DNA oligo hybridizing to the *ratA* mRNA was employed (top) and an ethidium bromide-stained gel was used as a loading control (bottom).

Next, as our expression system under endogenous promoter control was capable of restoring the wild-type colony size, we aimed to determine which RatA variant was synthesized under non-overexpression conditions. Therefore, we first harvested cells from unstressed, exponentially growing cultures and investigated the synthesis of RatA_fl_ or RatA_Δ14_ by western blot analysis. As indicated in Figure 4A, only a single protein migrating at the corresponding size of RatA_Δ14_ was observed when the wild-type *ratA* sequence was employed. It should be noted that a SignalP 6.0 prediction of signal peptides returned a high probability of a Sec/SPI signal peptide for the first 20 amino acids of the RatA N-terminus (Figure S3). Therefore, the observed protein has the following two possible origins: either translation initiates on the endogenous mRNA at the annotated first start codon to synthesize RatA_fl_ and the efficient processing of the signal peptide results in the observed truncated variant of RatA, or translation initiates at the Met14 codon inside of the annotated ORF to directly synthesize RatA_Δ14_. To differentiate between these two possibilities, we mutated both potential start codons in our endogenous promoter construct. If the observed protein is produced by the proteolytic cleavage of the potential signal peptide, we would expect a loss of the signal upon the mutation of the first start codon (M1S). On the other hand, if the protein originates from an initiation at the Met14 codon, we expect the loss of the signal upon the introduction of the M14I mutation. As shown in Figure 4A, no RatA protein is observed when the Met14 codon is mutated, whereas a weaker but still detectable signal is present in the Met1 mutation strain. These findings are in line with the observation that gene expression from the *ratA(M1S)* mutant plasmid is sufficient to complement the previously observed small-colony phenotype (Figure 3, horizontal blue stripes boxes), whereas gene expression from the *ratA(M14I)* mutant plasmid displays the same phenotype as the *ratA* deletion strain (Figure 3, diagonal blue stripes boxes). Thus, we concluded that in exponentially growing cells, only RatA_Δ14_ originating from Met14 is synthesized.

### 2.5. The ratA σ^70^ Promoter Is Partially Located Inside the Annotated ORF

Intriguingly, we observed a significantly reduced RatA_Δ14_ abundance when employing the Met1 mutant in our endogenous expression system (Figure 4A), even though translation initiates downstream on Met14. Therefore, we speculated that the observed differences in protein amounts can be explained by a difference in mRNA abundance. Thus, we performed northern blot analysis on the total RNA isolated under the same growth conditions (Figure 4B). While comparable mRNA levels were observed for the *ratA(M1S)*–*His* and *ratA(M14I)*–*His* transcripts, both were clearly reduced compared to the wild-type *ratA*–*His* mRNA. This lowered mRNA abundance is either due to a reduced mRNA stability or reduced mRNA transcription. Previously, no solid experimental evidence has been described for the promoter of the *ratA* gene. Therefore, to gain insights into the transcription regulation of *ratA*, we employed a promoter prediction tool and identified a potential σ^70^ sequence with the −10 box positioned inside of the annotated ORF (Figure S4A,B). Consistent with this prediction, using primer extension analysis, we identified the 5′-end of the *ratA* mRNA downstream of the −10 box (Figure S4C). These findings are in agreement with our own previously published transcriptomics data [13] and previously published results globally mapping transcription start sites (TSSs) for *E. coli* (Figure S5, [14]). Importantly, the altered sequence in the M1S mutation negatively influenced the promoter prediction score (Figure S4B), potentially explaining the reduced mRNA levels (Figure 4B). To further confirm these results, we introduced several silent mutations in the −10 box to reduce the promoter prediction score even further (Figure S4B). Accordingly, no *ratA* mRNA was detected (Figure 4B) and no RatA_Δ14_ was observed (Figure 4A). Therefore, we concluded that in unstressed, exponentially growing *E. coli* cells, RatA_Δ14_ is translated from an mRNA under σ^70^ transcriptional control that does not include the annotated Met1 codon.

### 2.6. Only RatA_Δ14_ Is Detected over Different Growth Phases Specifically Under Aerobic Conditions

**Figure 5:**
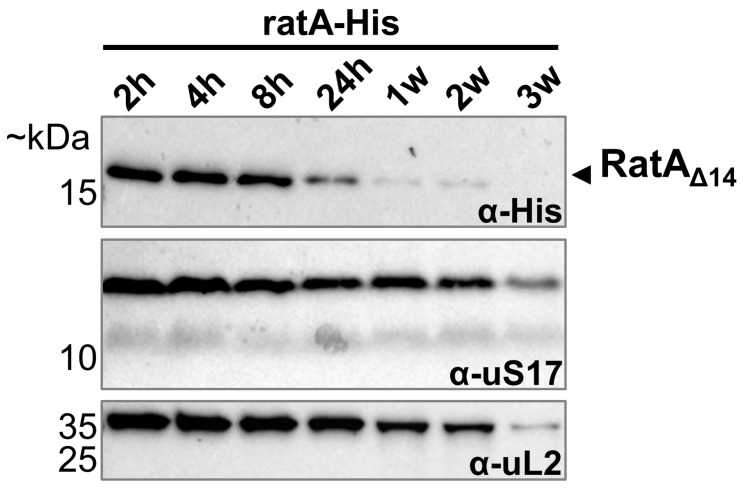
No RatA variant is synthesized in extended stationary phase. Strain BW25113 (Δ*ratA*) transformed with the low-copy plasmid pZS*2–ratA–His carrying the wild-type *ratA* gene with a 3′-terminal His-tag sequence under endogenous promoter control (ratA–His) was cultured at 37 °C in LB and samples were harvested for western blot analysis at several time points during the initial 24 h of growth (2 h–24 h), as well as after up to 3 weeks in extended stationary phase (1 w–3 w). RatA_Δ14_ (or RatA_fl_) levels were investigated using a primary antibody targeting the C-terminal His-tag (top). As a comparison, the protein levels of both a small (uS17) and a large ribosomal subunit protein (uL2) are shown. A reduction in ribosomal protein levels towards extended stationary phase is expected [15].

**Figure 6:**
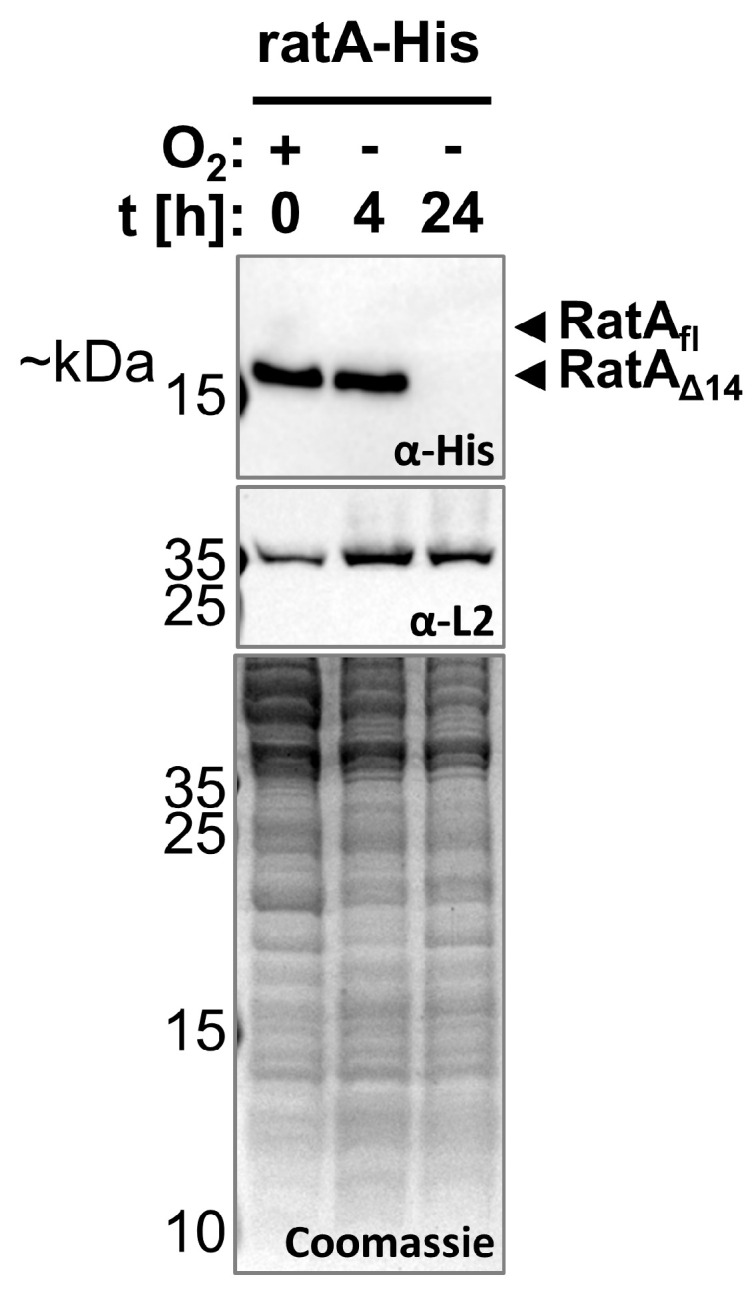
No RatA variant is present after 24 h of anaerobic growth. Strain BW25113 (Δ*ratA*) transformed with the low-copy plasmid pZS*2–ratA–His carrying the wild-type *ratA* gene with a 3′-terminal His-tag sequence under endogenous promoter control (ratA–His) was cultured overnight aerobically at 37 °C in LB and first samples (t = 0 h) were harvested for western blot analysis before resuspension of the cells in fresh, oxygen-deprived LB. Additional samples for western blot analysis were then harvested after 4 and 24 h of anaerobic cultivation at 37 °C. RatA_Δ14_ (or RatA_fl_) levels were investigated using a primary antibody targeting the C-terminal His-tag (top). As a comparison, the protein levels of the large ribosomal subunit protein (uL2) are shown and a Coomassie gel was used as an additional loading control (bottom).

So far, when *ratA* was under endogenous promoter control, we were only able to detect the RatA_Δ14_ variant and not the toxic RatA_fl_ variant. However, as mentioned above, the previous results were all obtained from cells sampled in the exponential phase. Therefore, the question remains of whether we can observe RatA_fl_ under different growth conditions. Thus, we monitored *ratA* expression from the early exponential phase to the extended stationary phase. As shown in Figure 5, the RatA_Δ14_ variant was readily detected during the first 8 h of growth, with the signal intensity steadily declining from 24 h to 2 weeks of culturing the bacteria. After 3 weeks in the stationary phase, no more RatA_Δ14_ was detectable. However, no RatA_fl_ was detected at any time point from the early exponential to the prolonged stationary phase (Figure 5). Thus, we concluded that the transition to the stationary phase does not induce the synthesis of RatA_fl_ and that the function of RatA_Δ14_ is related to active growth. Moreover, Fino et al. described PasT as a lipid chaperone for ubiquinone in the electron transport chain. Fittingly, they observed no small-colony phenotype when *pasTI* deletion cells were grown under anaerobic conditions [10]. Therefore, we decided to analyze the synthesis of RatA_Δ14_ (or RatA_fl_) in the absence of oxygen. As before, cells expressing the wild-type *ratA*–*His* gene under endogenous promoter control were grown aerobically overnight before being re-diluted and switched to anaerobic conditions. In contrast to cells cultured aerobically (Figure 5), in the absence of oxygen, no more RatA_Δ14_ was detectable after 24 h (Figure 6). Thus, these results indicate that RatA_Δ14_ is similarly involved in respiration, as has been described for PasT [10].

## 3. Discussion

### 3.1. The Toxic Effect of RatA Is an Artifact of Overexpressing an Incorrectly Annotated Gene

Previous studies describe RatA as the toxin moiety of a potential TA system, targeting the large subunit of the ribosome [5,7,8]. Several lines of evidence are in contrast to these findings. First, *ratA* is encoded in the bi-cistronic operon *ratAB*. However, in most type II TA pairs, the toxin moiety is encoded downstream of the antitoxin [6]. Furthermore, genomic deletions of *ratB* are viable [9], which argues against *ratAB* being a classical TA system. Lastly, Fino et al. found no deleterious effect on growth upon the overexpression of *ratA* in spot assays and instead described PasT, the UPEC homolog of RatA, as a lipid chaperone involved in the electron transport chain [10].

In this study, we determined that the previously described toxic effect of RatA is an artifact originating from the mis-annotation of the start codon of *ratA*. The overexpression of the coding sequence of *ratA* as it is currently annotated in the K-12 reference genome resulted in the synthesis of two RatA variants with differing lengths (Figure 1B) and varying effects on cells (Figure 1C and Figure 2). We showed that the toxic RatA_fl_ originates from the annotated start codon (Met1), whereas the shorter, non-toxic RatA_Δ14_ originates from translation initiation at Met14 (Figure 1D). Unlike the findings of Zhang and Inouye [7], we did not observe a distinct association of RatA_fl_ with the 50S subunit (Figure 2).

To determine which variant of RatA is synthesized in vivo, we employed an endogenous promoter system under aerobic conditions. Independent of the growth phase, only RatA_Δ14_ was synthesized in *E. coli* cells (Figure 4 and Figure 5). Additionally, we identified a promoter sequence for *ratA* that positions the TSS downstream of the Met1 codon (Figure 4 and Figure S4), further confirming that translation initiation occurs at the Met14 codon. The promoter sequence identified is close to the consensus sequence associated with the housekeeping sigma factor σ^70^ (Figure S4). This aligns with the proposed role of PasT as a lipid chaperone involved in respiration [10], which is further supported by our findings that no RatA_Δ14_ was detected after 24 h of growth under anaerobic conditions (Figure 6). However, we cannot completely exclude the possibility that a second promoter could be located upstream of Met1 that relies on different σ factors (e.g., the general stress response sigma factor [16]) or requires the activation of certain transcription factors as a response to specific stress conditions (e.g., OxyR as a response to oxidative stress [17]). In theory, it is therefore still possible that an mRNA originating from a promoter further upstream could be transcribed in the wild-type *E. coli* strain, but based on the results presented here, we concluded that RatA_fl_ was of no physiological relevance under the tested conditions.

### 3.2. False Annotations of the Initiating Start Codon Could Be More Widespread

As described above, our data show that the ORF for *ratA* was incorrectly annotated. Previous studies in other bacterial model organisms have observed similar discrepancies in up to 7% of ORFs [18], suggesting that this might be a more widespread problem and not restricted to this specific gene and species. In 1997, when the complete genome sequence of the first *E. coli* K-12 strain was published [2], Blattner et al. were tasked with assigning the N-termini of all identified ORFs. However, most ORFs contain multiple in-frame methionine codons and many of these genes still remain uncharacterized today. Therefore, if no additional information was available, the authors generally chose to select the longest possible ORF to “preserve the most coding information for analysis” [2]. While the authors did warn that this may not reflect the situation in vivo, as evidenced by the history of *ratA*, this warning may have been lost in the course of time.

Therefore, we analyzed all protein sequences deposited in the UniProt Knowledgebase (UniProtKB) for the *E. coli* strain K-12 (Taxonomy ID: 83333). Out of 4587 entries, 1055 contain at least one additional, in-frame methionine codon at the amino acid positions 2–15. From these 1055 ORFs, 255 proteins remain uncharacterized or are assigned only a putative function (Table S1). Furthermore, previous publications to globally determine the TSSs of *E. coli* have often found a TSS inside of the annotated ORF. In a first approach in 2009, Mendoza-Vargas et al. described 21 ORFs where the TSS was found within the coding region [19]. Notably, *ratA* (or formerly *yfjG*) was not among them, indicating that this might be a more widespread phenomenon than that identified by Mendoza-Vargas et al. Indeed, in a later study applying a differential RNA sequencing approach, out of 14,868 TSS candidates, Thomason et al. internally identified 5574 TSS candidates of annotated genes [14]. While many of these candidates accumulated at the 3′-end of the respective genes, a significant portion was still found within the first 10% of the gene sequence. Among them, as mentioned above, was *ratA* (Figure S5). Therefore, taken together, this study highlights the importance of first validating the basics of molecular biology when investigating a previously poorly described gene, even in times of advanced high-throughput techniques.

## 4. Materials and Methods

### 4.1. Bacterial Strains and Plasmids Construction

The wild-type *E. coli* K-12 strain BW25113 and the corresponding genomic deletion of *ratA* were obtained from the Keio collection (JW2600, [9]). The excision of the kanamycin resistance cassette was performed using the heat-sensitive plasmid pCP20, as described in the original publication [20].

For detailed information on the primers employed in this study for the construction of the individual plasmids, see Table S2. Generally, the genomic region of interest was amplified by PCR (Thermo Scientific (Waltham, MA, USA), 2× Phusion Master Mix with HF Buffer) from genomic DNA (gDNA) and subsequently digested with appropriate restriction enzymes using the FastDigest restriction enzymes system from Thermo Scientific. Digested fragments were then ligated (Thermo Scientific (Waltham, MA, USA), T4 DNA Ligase) into corresponding, equally digested vectors. Point mutations and the addition of 3′-terminal His-tag sequences were introduced by inverse PCR followed by blunt-end ligation.

For the arabinose inducible overexpression of *ratA*, the complete ORF of the wild-type *ratA* gene as currently annotated in the NCBI reference sequence NC_000913.3 was inserted into the pBAD33 empty vector [21] (see also Figure S1A). No promoter has previously been experimentally confirmed for *ratA*. Therefore, to retain as many potential regulator elements as possible for the simulated endogenous promoter expression of *ratA*, the complete intercistronic region between *ratA* and the upstream *smpB* gene, as well as the region encoding for the first 46 amino acids of *smpB*, was inserted into the low-copy plasmid pZS*2 [22] (see also Figure S1A). To prevent secondary effects from a potential SmpB-fragment, a degradation tag was added in frame to the partial *smpB* gene to ensure efficient degradation.

### 4.2. Bacterial Culturing and Growth Conditions

Glycerol stocks of the constructed strains were routinely plated on LB+agar plates (10 g/L of peptone, 5 g/L of yeast extract, 10 g/L of NaCl, 1.5% *w*/*v* agar) and incubated overnight at 37 °C. Single colonies grown on the plates were then pre-cultured overnight in LB medium at 37 °C, 165 rpm before being re-diluted in fresh medium to an OD_600_ of 0.05 and incubated in the same conditions, with the growth being monitored by measuring the OD_600_ absorption. If required for plasmid retention, 30 µg/mL of chloramphenicol or 25 µg/mL of kanamycin were supplemented in the medium. For the induction of *ratA* overexpression from the pBAD–ratA plasmid, arabinose was added to the culture to a final concentration of 0.2% (*w*/*v*). For anaerobic growth, an aliquot of the cells from the aerobically grown overnight pre-culture was harvested by centrifugation at 3000× *g* for 2 min at room temperature, transported to an anaerobic chamber, and resuspended in an equal volume of nitrogen-flushed LB medium before being 100-fold diluted in nitrogen-flushed LB medium and incubated at 37 °C. For each described individual growth experiment, at least three biological replicates were performed.

### 4.3. Colony Size Determination

For determining the average colony size, the different strains were pre-cultured overnight in LB + 25 µg/mL of kanamycin as described above and re-diluted in fresh medium to an OD_600_ of 0.05. The resulting culture was further serially diluted to a final dilution factor of 10^−5^ before plating 50 µL on LB plates with antibiotic added for plasmid retention. All plates were incubated at 37 °C for 20 h to allow for the growth of single colonies. From the resulting plates, an image was taken and the sizes of 50 individual colonies were measured with Fiji [23].

### 4.4. Western Blot Analysis

At the indicated time points, the OD_600_ of the individual bacterial cultures was measured and samples were harvested at 10,000× *g* for 2 min at room temperature. The supernatant was removed and 2× Laemmli buffer (4% SDS, 20% glycerol, 0.004% bromophenol blue, 125 mM Tris base, 10% β-mercaptoethanol, pH 6.8) was added to the pellet to a final calculated concentration of 20 OD units per mL. Afterwards, cells were directly opened and proteins were denatured simultaneously by cooking the resuspended pellet at 85 °C for 5 min. In total, 0.1 OD units were loaded per sample and proteins were separated on 16% tris-tricine gels. For loading control, a second gel was regularly prepared for Coomassie staining of the proteome. For the detection of individual proteins, a wet transfer to 0.2 µm nitrocellulose membranes (Amersham Protran, Cytiva Marlborough, MA, USA) was performed at 100 V for 1 h at 4 °C in 1× transfer buffer (25 mM Tris base, 192 mM Glycine, pH 8.3). Membranes were blocked with 5% milk powder in 1× PBS supplemented with 1% Tween-20 (1× PBS-T) before probing with anti-His primary antibody (Cytiva 27471001, raised in rabbit) overnight at 4 °C. The next day, anti-rabbit IgG coupled to horseradish peroxidase (Cell Signaling Technology 7074S, Cell Signaling Technology, Danvers, MA, USA) was used as a secondary antibody. For r-proteins, lab stocks of primary antibodies raised in goat were employed in combination with anti-goat IgG (Sigma Aldrich A5420, Merck, Darmstadt, Germany). All blots were developed using the SuperSignal West Pico PLUS Kit chemiluminescent reagent from Thermo Scientific (Waltham, MA, USA) in the BioRad (Hercules, CA, USA) ChemiDoc Imaging system.

### 4.5. Sucrose Gradient Analysis

The overexpression of *ratA–His* and the corresponding mutants was induced in exponentially growing cells with 0.2% arabinose for 1.5 h before increasing the chloramphenicol concentration to 200 µg/mL and pouring 150 mL of each culture over 100 mL or frozen 1× PBS. The cells of the cooled down cultures were then harvested at 10,000× *g* for 10 min at 4 °C before being resuspended in cold 1× TMN buffer (10 mM Tris, 10 mM MgCl_2_, 50 mM NH_4_Cl, 6 mM β-mercaptoethanol, pH 7.6) supplemented with 200 µg/mL of chloramphenicol, 1 mg/mL of lysozyme, and 1 U/mL of DNase I. The cells were then lysed using several freeze and thaw cycles. The resulting cell debris was removed by centrifugation at 30,000× *g* for 15 min at 4 °C. Of the collected supernatant, 10 A_260_ units were loaded onto a 10–40% sucrose gradient in 1× TMN buffer and centrifuged at 34,000 rpm for 3 h at 4 °C using a SW40 Ti rotor. Following centrifugation, the gradients were fractionated using a piston gradient fractionator (Biocomp Instruments, Fredericton, NB, Canada), collecting 13 fractions of 930 µL each while monitoring the absorbance at 254 nm. The proteins in the collected fractions were precipitated by the addition of 190 µL of 100% trichloroacetic acid to each fraction, followed by incubation on ice for 1 h. The precipitated proteins were collected at 18,000× *g* for 15 min at 4 °C and washed with 400 µL of 80% acetone solution in water. The final pellets were air-dried and resuspended in 2× Laemmli buffer for western blot analysis, as described above.

### 4.6. Northern Blot and Primer Extension Analysis

In total, 50 mL of exponentially growing cells was harvested at 10,000× *g* for 4 min at 4 °C and the total RNA was extracted from the resulting pellet using 1 mL of TRIzol reagent from Invitrogen (Waltham, MA, USA) according to the manufacturer’s instructions. A total of 10 µg total RNA each was then separated on a 7% polyacrylamide gel (29:1 acrylamide/bis-acrylamide) in 1× TBE (100 mM Tris, 100 mM boric acid, 2 mM EDTA, pH 8.0) supplemented with 8 M urea. The resulting gel was stained with ethidium bromide as a loading control before the transfer of the RNA onto an Amersham Hybond-N+ nylon membrane from Cytiva (Marlborough, MA, USA) using a semi-dry blotting system with 0.5× TBE buffer. After the transfer, the RNA was crosslinked to the membrane by UV light exposure before probing with the single-stranded DNA oligo IM_O80 (5′-GTTACTGGTCAACTGGTTGCG-3′) that was ^32^P-labeled at its 5′-end using the T4 polynucleotide kinase system from Thermo Scientific (Waltham, MA, USA). Hybridization occurred overnight at 48 °C in ROTI^®^Hybri-Quick buffer from ROTH (Karlsruhe, Germany). Afterwards, washed membranes were exposed in storage phosphor screens and the resulting image was analyzed using the Typhoon FLA 9500 phosphorimaging device from GE Life Sciences (Chicago, IL, USA)

For primer extension analysis, 5 µg of isolated total RNA was used as an input for complementary DNA synthesis using the SuperScript III reverse transcriptase system from Invitrogen (Waltham, MA, USA), with the ^32^P-labeled single-stranded DNA oligo IM_W80 (5′-TAACTGATACATTTGCTCCGCGC-3′) being used as a primer for the first-strand synthesis. For sequencing lanes, in vitro transcribed RNA from the corresponding genomic region was used as an input and reverse transcription was performed with the AMV reverse transcriptase from Promega (Madison, WI, USA) with the corresponding ddNTP supplemented in the individual sequencing reactions. After reverse transcription, the resulting complementary DNA fragments were separated on an 8% polyacrylamide gel (19:1 acrylamide/bis-acrylamide) in 1x TBE supplemented with 8 M urea before the gel was dried onto a Whatman 3 MM CHR paper from Cytiva (Marlborough, MA, USA) and exposed for phosphorimaging, as described above.

## Data Availability

The original contributions presented in this study are included in the article and Supplementary Materials. Further inquiries can be directed to the corresponding authors.

## Supplementary Materials

The following supporting information can be downloaded at: https://www.mdpi.com/article/10.3390/ijms26073100/s1. References [24,25] are cited in Supplementary Materials.

## Abbreviations

The following abbreviations are used in this manuscript:

NCBI	National Center for Biotechnology Information
RatA	Ribosome association toxin A
TA	Toxin–antitoxin
UPEC	Uropathogenic *E. coli*
ORF	Open reading frame
TSS	Transcription start site
UniProtKB	UniProt Knowledgebase
gDNA	Genomic DNA
