# Supplementary material for: Lessons from RatA: Why the Basics in Molecular Biology Are Still Crucial!
Source: Int J Mol Sci. 2025 Mar 27;26(7):3100. doi: 10.3390/ijms26073100 (PMC11988751; doi:10.3390/ijms26073100)
Supplement: Supplementary file 1 [file ijms-26-03100-s001.zip › Supplementary Data - Fasnacht et al. 2025.pdf]

Supplementary Data

Lessons from RatA: Why the basics in molecular biology are still crucial!

Michel Fasnacht [1,2,*], Denise Schratt [1,2] and Isabella Moll [1,2,*]

[1] Max Perutz Labs, Vienna Biocenter Campus (VBC), Dr.-Bohr-Gasse 9 / Vienna Biocenter 5, 1030, Vienna, Austria.
[2] University of Vienna, Max Perutz Labs, Department of Microbiology, Immunobiology and Genetics Dr.-Bohr-Gasse 9 / Vienna Biocenter 5, 1030, Vienna, Austria.
* Correspondence: MF michel.fasnacht@univie.ac.at, IM isabella.moll@univie.ac.at

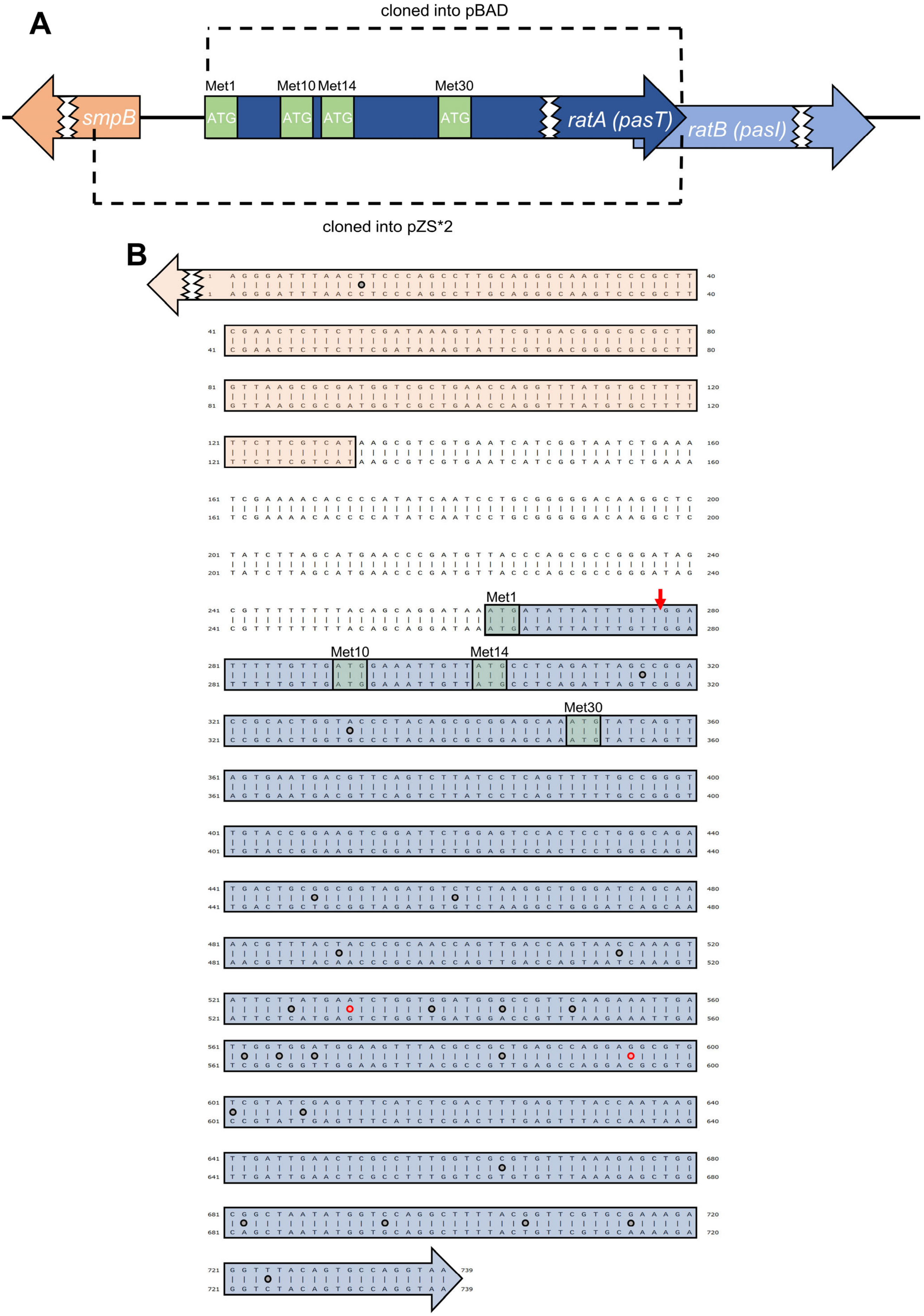
A
cloned into pBAD
Met1
Met10
Met14
Met30
ATG
ATG
ATG
ATG
smpB
ratA (pasT)
ratB (pasI)
cloned into pZS*2
B
Met1
Met10
Met14
Met30

Figure S1. **Genomic locus and sequence alignment *ratAB/pasTI***

A Not-to-scale representation of the genomic locus surrounding the *ratAB* (or *pasTI* in CFT073) operon as currently annotated in the K-12 reference genome. Different in-frame methionine codons at the 5′-end of the *ratA* gene are indicated in green. The corresponding genomic regions that were cloned into the pBAD plasmid for overexpression or into the pZS*2 plasmid for endogenous promoter control expression are marked. **B** For the larger genomic region cloned into pZS*2, a DNA sequence alignment was performed between the corresponding regions of the BW25113 reference genome (top strand, GenBank CP009273.1) and CFT0713 reference genome (bottom strand, GenBank AE014075.1). Silent mutations are marked by black circles, non-silent mutations by red circles. The transcription start site of the *ratA* mRNA as identified by this study is marked by a red arrow.

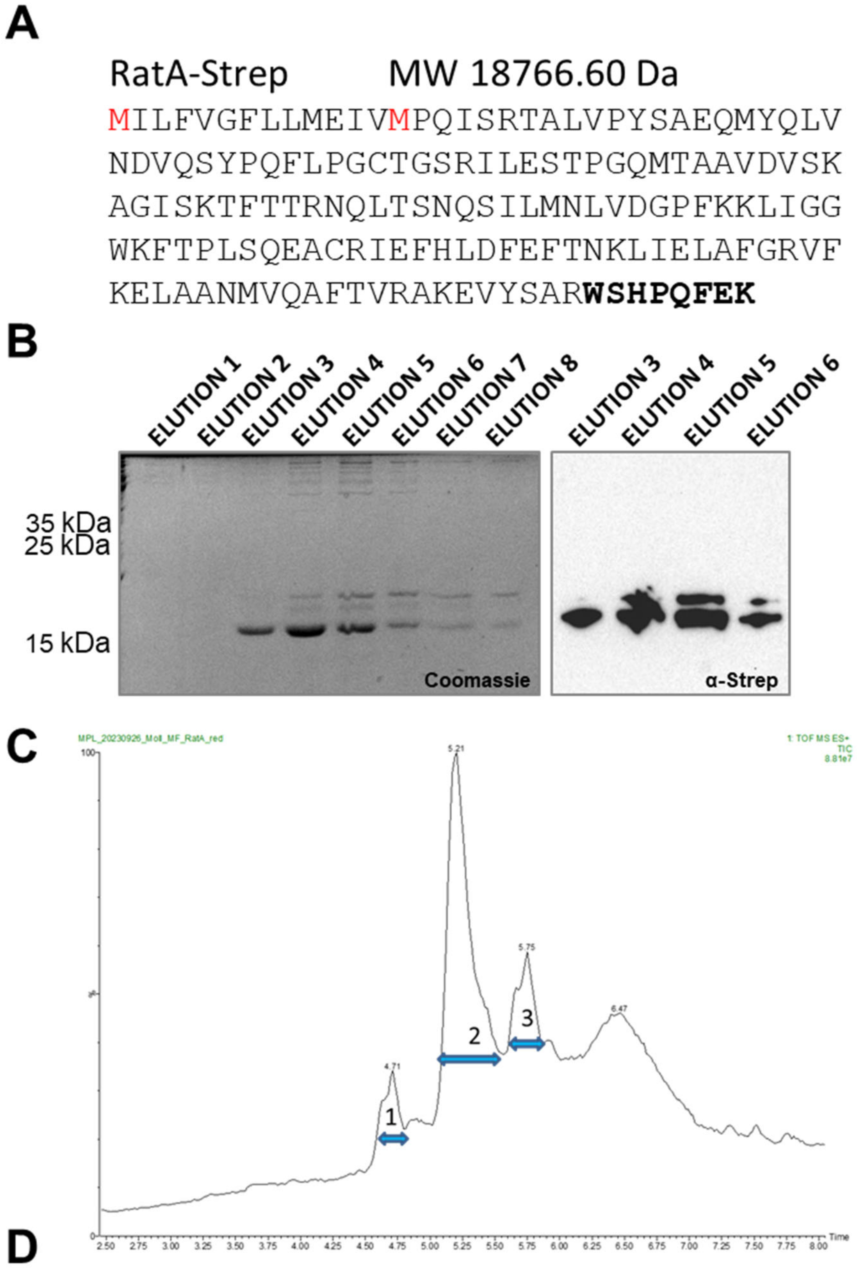

D

Peak	Experimental masses (Da)	Interpretation	Mass error (Da)
1	16912.0	?	
	68984.8	dnaK, N-term Met excised	+ 1.4
2	17128.0	RatA, AA 15-166	- 0.5
3	17127.9	RatA AA 15-166	- 0.5
	18766.2	RatA, FL	- 0.4
	21666.2	?	
	57197.3	groEL, N-term Met excised	+ 0.3

Figure S2. Intact mass determination of both RatA variants by LC-MS

A Sequence and calculated molecular weight of RatA-Strep. Met1 and Met14 are labelled in red, the C-terminal Strep tag is indicated in bold. **B** Coomassie stain of different elution fractions for the purification via Strep-beads of the two RatA-Strep variants and the corresponding western blot of fractions 3-6. Elution 6 was sent for intact mass determination by LC-MS. **C** Chromatogram (total ion current plotted against time) of elution 6. Indicated peaks were further analyzed by TOF-MS with the resulting reconstructed average masses of detected proteins summarized in **D**. Wherever possible, proteins were assigned by comparison of the calculated and experimentally determined mass. Question marks indicate that no catalogued *E. coli* protein could be matched.

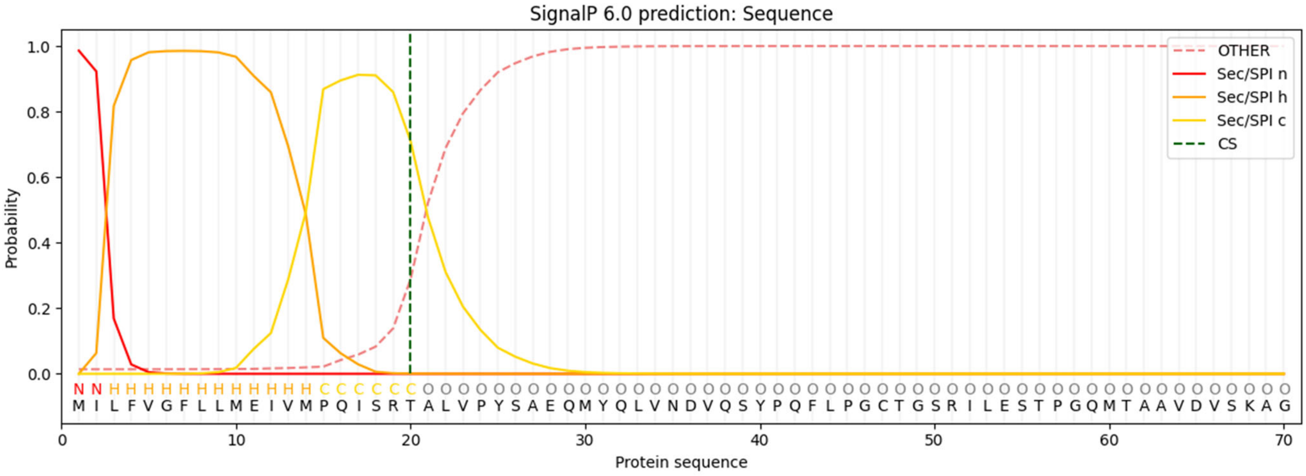

Figure S3. SignalP 6.0 prediction [24] reveals a potential membrane targeting sequence at the N-terminus of RatA with the cleavage site (CS) after the methionine at position 14.

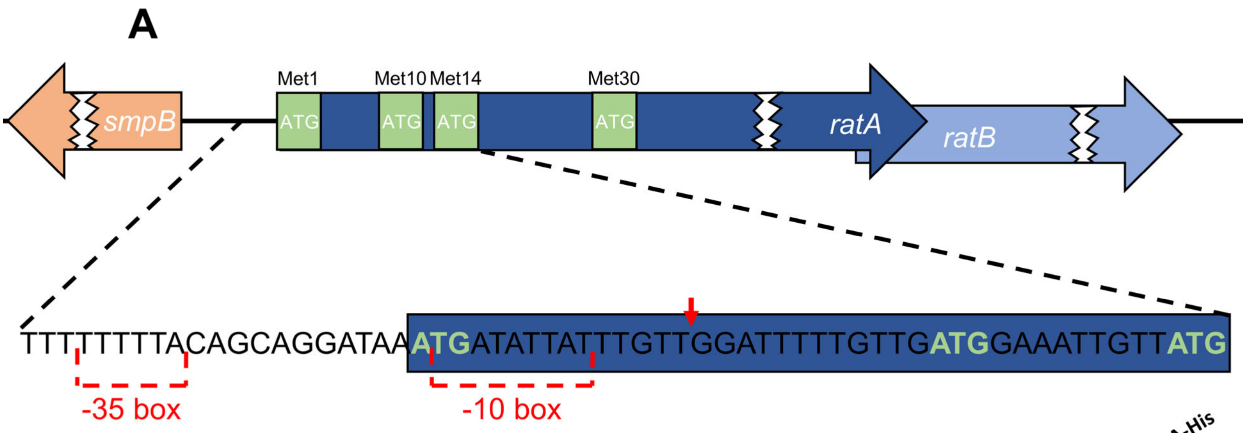

B

Mutation	Sequence	BPROM Score
wildtype	**ATG**ATATTAT	68
M1S	**A**GCATATTAT	51
-10 box	**ATG**ATCCTTT	8

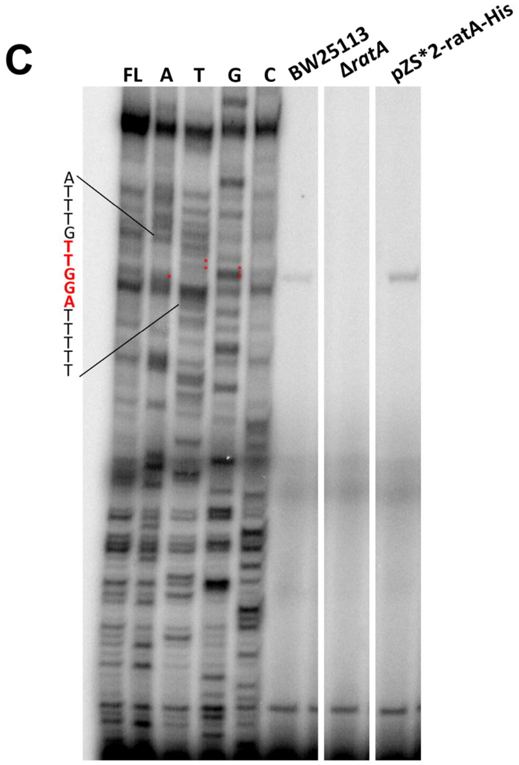

Figure S4. A σ[70] promoter sequence for *ratA* is located partially inside the annotated ORF

A The -35 box and -10 box sequences as predicted by BPROM [25] are indicated in the blowup of the sequence representing the K-12 genomic region upstream of the annotated start codon of *ratA* to the Met14 codon. The corresponding transcription start site as identified by this study is marked by a red arrow. **B** Predicted -10 box sequences and their respective BPROM scores of the corresponding predicted σ[70] promoter are shown for the wildtype *ratA* sequence, the M1S mutation, and the -10 box mutation sequence containing several silent mutations compared to the wildtype sequence. **C** Primer extension analysis of total RNA isolated from unstressed, exponentially growing cells identifies a transcription start side inside of the annotated open reading frame independent of whether *ratA* was encoded in the genome of the wildtype BW25113 strain or expressed from a plasmid encoded copy on the pZS*2-ratA-His plasmid in the BW25113(Δ*ratA*) strain. As a negative control, no corresponding primer extension stop is identified in the BW25113(Δ*ratA*) strain.

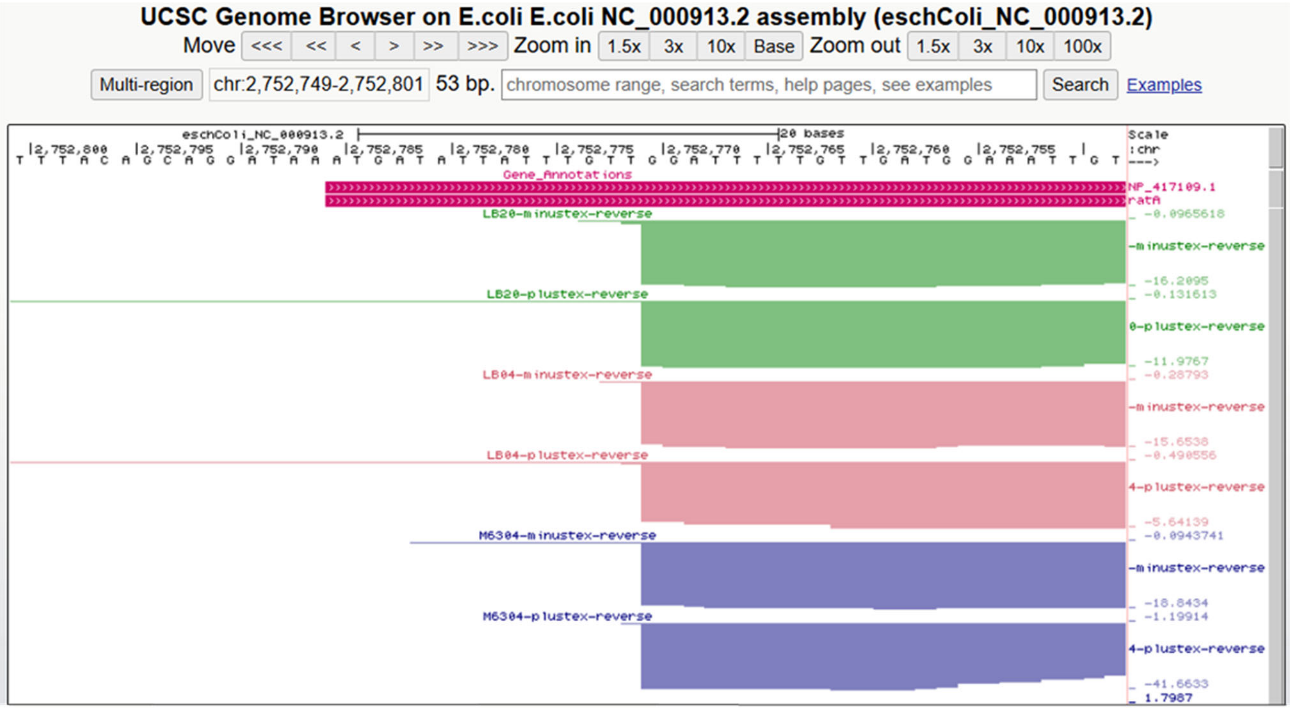

Figure S5. Transcriptomic approaches identify the same transcription start site.
A UCSC genome browser screenshot of differential RNA sequencing to identify transcription start sites of *E. coli* reveals a transcription start site inside of the annotated open reading frame of *ratA* [14]. The same 5´-end of the *ratA* transcripts was identified in our own previously published RNA sequencing data of exponentially growing, unstressed cells [13].

Supplemental Materials and Methods

Intact mass determination by LC-MS

The two RatA-Strep protein variants were ectopically produced by overexpression from the pBAD-ratA-Strep plasmid in the *E. coli* K-12 strain BW25113 and purified using Strep-Tactin® Sepharose beads from IBA Lifesciences (Art. Nr. 2-1201-010) according to the manufacturer´s protocol. Different elution fractions were analyzed first by Coomassie staining of the eluted proteins, followed by western blot confirmation using the Strep-tag II antibody (PK-AB718-4217). Intact mass of the eluted proteins was determined by LC-MS. Therefore, protein samples were diluted in water to 4 ng/µL and dithiothreitol was added to a final concentration of 25 mM. Eight nanogram (2 µL) were loaded on an XBridge Protein BEH C4 column (2.5 µm particle size, dimensions 2.1 mm X 150 mm; Waters) using a Dionex Ultimate 3000 HPLC system (Thermo Scientific) with a working temperature of 50 °C, 0.1% formic acid (FA) as solvent A, 100% acetonitrile, 0.08% FA as solvent B. Proteins were separated on a 6 min step gradient from 12 to 72% solvent B at a flow rate of 250 µL/min and analyzed on a Synapt G2-Si coupled via a ZSpray ESI source (Waters). Data were recorded with MassLynx V 4.2 (Waters) and analyzed using the MaxEnt 1 process to reconstruct the uncharged average protein mass.

References

13. Sauert, M.; Wolfinger, M.T.; Vesper, O.; Müller, C.; Byrgazov, K.; Moll, I. The MazF-Regulon: A Toolbox for the Post-Transcriptional Stress Response in *Escherichia Coli*. *Nucleic Acids Research* **2016**, *44*, 6660–6675, doi:10.1093/nar/gkw115.
14. Thomason, M.K.; Bischler, T.; Eisenbart, S.K.; Förstner, K.U.; Zhang, A.; Herbig, A.; Nieselt, K.; Sharma, C.M.; Storz, G. Global Transcriptional Start Site Mapping Using Differential RNA Sequencing Reveals Novel Antisense RNAs in *Escherichia Coli*. *J Bacteriol* **2015**, *197*, 18–28, doi:10.1128/JB.02096-14.
24. Teufel, F.; Almagro Armenteros, J.J.; Johansen, A.R.; Gíslason, M.H.; Pihl, S.I.; Tsirigos, K.D.; Winther, O.; Brunak, S.; von Heijne, G.; Nielsen, H. SignalP 6.0 Predicts All Five Types of Signal Peptides Using Protein Language Models. *Nat Biotechnol* **2022**, *40*, 1023–1025, doi:10.1038/s41587-021-01156-3.
25. Solovyev, S.V.; Salamov, A.; Li, R.W. *Automatic Annotation of Microbial Genomes and Metagenomic Acts. Gen Metagenomics and Its Applications in Agriculture, Biomedicine and Environmental Studies*; Nova Science Publishers. Hauppauge, NY, USA, 2011;
